# CPT-SIOP registry: evaluation of risk factors for disease progression in pediatric choroid plexus papilloma

**DOI:** 10.1007/s11060-025-05136-4

**Published:** 2025-06-26

**Authors:** Christian Fiedler, Uwe R. Kordes, Christian Thomas, Martin Hasselblatt, Brigitte Bison, Lars Behrens, Martin Mynarek, Rolf-Dieter Kortmann, Beate Timmermann, Torsten Pietsch, Jenny Adamski, Johannes E A Wolff, Stefan Rutkowski, Denise Obrecht-Sturm

**Affiliations:** 1 Department of Pediatric Hematology and Oncology, University Medical Center Hamburg-Eppendorf, Hamburg, Germany; 2 Institute of Neuropathology, University Hospital Münster, Münster, Germany; 3 Department of Diagnostic and Interventional Neuroradiology, University Hospital Augsburg, Augsburg, Germany; 4 Department of Radiation Oncology, University of Leipzig, Leipzig, Germany; 5 Department of Particle Therapy, West German Proton Therapy Centre Essen (WPE), University Hospital Essen, West German Cancer Center (WTZ), German Cancer Consortium (DKTK), Essen, Germany; 6 Department of Neuropathology, Deutsche Gesellschaft für Neuropathologie und Neuroanatomie (DGNN) Brain Tumor Reference Center, Bonn, Germany; 7 Mildred Scheel Cancer Career Center HaTriCS4, University Medical Center Hamburg-Eppendorf, Hamburg, Germany; 8 Birmingham Women’s and Children’s Hospital NHS Foundation Trust, Birmingham, UK; 9 Department of Pediatrics, The University of Texas M. D. Anderson Cancer Center, Unit 87, Houston, TX USA

**Keywords:** Choroid plexus tumor, Choroid plexus papilloma, Atypical choroid plexus papilloma

## Abstract

**Purpose:**

Choroid plexus papilloma (CPP) and atypical choroid plexus papilloma (aCPP) have excellent outcomes. However, some CPP/aCPP relapse and may qualify for postoperative adjuvant treatment.

**Methods:**

German patients from the International CPT-SIOP Registry diagnosed with CPP/aCPP between 2011 and 2023 were included and analysed according to initial staging (postoperative residual tumor [R+], metastases [M+]), biology, postoperative treatment strategy and outcome. Additionally, patients from the published CPT-SIOP-2000 trial (PMID34997889) were combined with the registry cohort for validation purpose.

**Results:**

Ninety-three patients were identified (male: n = 53, female: n = 40). Median age at diagnosis was 1.9 (0.1–17.6) years. Initial staging was R0/M0 in n = 61, R+/M0 in n = 24, R0/M + in n = 5 and R+/M + in n = 3. aCPP was diagnosed in n = 38 patients. Molecular subgroup was available for n = 36: ”adult” *n* = 3, “pediatric A” *n* = 21 and “pediatric B” *n* = 12 (6/12 aCPP). Median follow-up was 5.5 (± 0.99) years. Twelve tumors relapsed: R0/M0 *n* = 4, R+/M0 *n* = 7, R+/M + *n* = 1. One patient with relapse died. Most patients did not receive postoperative treatment (*n* = 88). Five patients (R0/M + *n* = 2; R+/M + *n* = 1; R0/M0 *n* = 2) received postoperative chemotherapy. None was irradiated during first-line treatment. In the enlarged cohort (*n* = 197), histological diagnosis had a significant impact on PFS (5y-PFS: CPP 90 ± 3.1, aCPP 78.6 ± 4.6, PFS = 0.01). Both, R+ (5y-PFS: R0 90.6 ± 2.6, *R* + 69.1 ± 7.0, PFS = 0.01) as well as molecular subgroup “pediatric B” (5y-PFS: pediatric A 95.2%±3.3%, pediatric B: 69.5 ± 8.6%, PFS = 0.02), were associated with inferior PFS, especially in aCPP.

**Conclusion:**

Incomplete resection and biology impact on PFS especially in aCPP. These results extend the evidence for current stratification and treatment strategies.

**Supplementary Information:**

The online version contains supplementary material available at 10.1007/s11060-025-05136-4.

## Introduction

Tumors originating from the epithelium of the choroid plexus (choroid plexus tumors, CPT) represent a rare brain tumour entity, primarily affecting young patients with median age at diagnosis of 2.1 years [1]. CPT account for 2–4% of all paediatric brain tumours in the first year of life and rank among the most common CNS tumors in this specific age group [2–4]. 

CPT are further divided into three different histological subtypes according to the 2021 WHO classification [5]: benign, low-grade choroid plexus papillomas (CPP, WHO grade 1), intermediate-grade atypical plexus papillomas (aCPP, WHO grade 2) defined by presence of increased mitotic activity and high-grade choroid plexus carcinomas (CPC, WHO grade 3) characterized by frank signs of malignancy, including brisk mitotic activity, nuclear pleomorphism and necrosis [5, 6]. Based on genome-wide DNA methylation-profiling, molecular subgroups can be differentiated: supratentorial, low-risk paediatric tumors (histological CPP and aCPP; “pediatric A”), supratentorial, high-risk pediatric tumors (histological CPP, aCPP and CPC; “pediatric B”) and infratentorial, low-risk tumors predominantly effecting adult patients (histological CPP and aCPP, “adult”) [7, 8]. 

CPC are notable for their pronounced incidence of *de novo Li-Fraumeni* Syndrome (LFS) and somatic *TP53* mutations [9]. Nevertheless, the majority of CPP do not exhibit *TP53* mutations, suggesting the presence of alternative underlying events in tumorigenesis, that are still unclear and under investigation [7]. Clinically, patients with CPP/aCPP have favorable long-term outcomes. However, relapses and dissemination along the neuroaxis may occur [1, 10, 11]. Especially aCPP is linked to a higher risk for relapse particularly in older individuals (≥ 3 years) and adults, while Wolff et al. showed that progression-free survival (PFS) and overall survival (OS) are superior for aCPP patients who were < 2 years of age at diagnosis [1, 12, 13]. 

Despite these findings, predicting relapse in individual patients with CPP or aCPP remains challenging. The prognostic relevance of molecular subgroup status, residual tumor and/or metastasis at initial diagnosis on the subsequent risk of relapse is still debated in the literature.

Identifying patients who are likely to benefit from adjuvant therapy, while sparing others from unnecessary treatment, remains a critical clinical goal.

The aim of this study is to report a large series of CPP/aCPP and analyse the impact of potential high-risk characteristics on the patients` outcome in an enlarged combined cohort to better define the risk for relapse and treatment indication in pediatric CPP and aCPP. These findings will help to guide future therapy decisions for this very rare patient population.

## Methods and materials

### Study design and patient selection

This retrospective analysis examines an unpublished patient cohort from the *International CPT-SIOP Registry* diagnosed with aCPP or CPP in Germany (registry cohort). Ethics approval was obtained by the competent authorities (Regensburg Nr 03/096, Hamburg PV3520). Patients and/or their legal guardians provided written informed consent for data collection, adhering to national law. Patients from the *International CPT-SIOP Registry* were included to this study if they fulfilled the following criteria: (A) histologically-confirmed newly-diagnosed aCPP or CPP, (B) available central pathology review, (C) age at initial diagnosis (time-point of first tumor resection or biopsy) from birth to 18 years and (D) initial diagnosis between 01.01.2011 to 01.01.2023. 197 patients were screened for this analysis. Details of the cohort are reported in Supplemental Table 1 “clinical courses”.

**Fig. 1:**
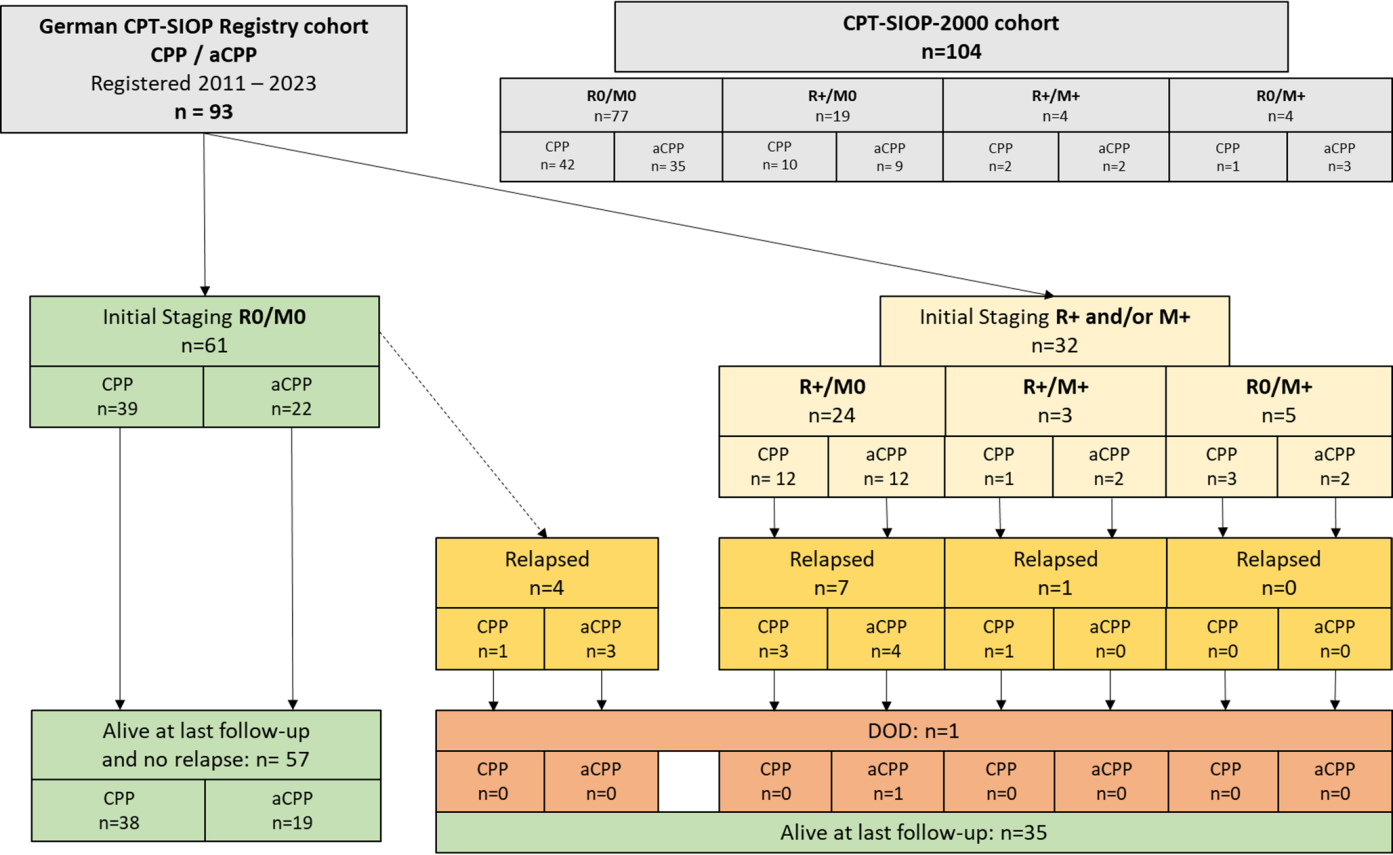
Consort diagram displaying the composition of the study cohort and the outcome of respective patients

Additional patients from the CPT-SIOP-2000 trial previously published by Wolff et al. (PMID: 34997889) diagnosed with CPP/aCPP were combined with the registry cohort to validate the observations and enable more robust statistical analyses [1]. In the following, this combined cohort is referred to as “enlarged cohort”. The cohort composition as well as an overview of the initial staging and outcomes are displayed by Fig. 1.

### Standard diagnostics and treatment

During initial work-up, central review of preoperative cranial and spinal MRI as well as lumbar puncture and cerebrospinal fluid (CSF) cytology were recommended but not mandatory. After tumor resection, a postoperative MRI within 24–72 h should be performed and central reviewed. Evaluation of lumbar cerebrospinal fluid (CSF) obtained on postoperative day 14 or later, but prior to the start of the adjuvant therapy, was recommended to identify tumor cells. Staging was subsequently classified as “R0/M0” (no evidence of residual tumor or metastases), “R+/M0” (residual tumor of any size, no evidence of metastases), “R0/M+” (metastases, no evidence of residual tumor) or “R+/M+”. Postoperative treatment strategy was determined on the treating physician`s decision, aligned with the national guidance following the CPT-SIOP-2000 trial protocol. During the period of this study, the following recommendation was used in Germany: in case of R+, evaluation of re-surgery was recommended. Further, patients with either non-resectable residual tumor or metastases were recommended to receive adjuvant treatment using Carb/EV chemotherapy as published by Wolff et al., while the remainder went into surveillance (“watchful waiting”) [1]. Follow-up MRI were recommended after each treatment element or every three months during the first year of follow-up and intervals were extended over the years. Response was measured analogously to the RANO/RAPNO criteria for medulloblastoma [14]: complete response (CR), with no tumor left/with evidence of complete tumor disappearance, partial response (PR) including all patients with tumor reduction of 50% or more of the largest measurable without reaching CR. Stable disease (SD) includes all patients whose tumor has shrunk by less than 50%, remained unchanged or has grown by less than 25% of the largest measurable diameter. Progressive disease (PD) was defined as tumor growth by 25% or more of the largest measurable diameter.

### Statistical analysis

IBM© SPSS© Version SPSS Statistics 29.0.1.0 was used for statistical analyses. Kaplan–Meier method estimated survival. OS was calculated from time of first tumor surgery until death from any cause. PFS was calculated from time of first tumor surgery to disease progression, relapse or death from due to tumor progression. Patients were censored at last follow-up. Uni- and multivariate cox regression models were used to evaluate potential independent risk factors.

For the Cox regression in the enlarged aCPP cohort, we excluded one value (“adult”) of the variable *molecular subgroup* due to the low number of cases (*n* = 1) to prevent statistical interference. To identify survival differences between subcohorts log-rank test with significance level set to 0.05 was used.

## Results

### German CPT-SIOP registry study cohort description

We identified ninety-three patients who match the inclusion criteria for this study, comprising 53 males and 40 females. Initial staging was R0/M0 in 61 patients (65.6%). We detected 32 patients (34.4%) with R + and/or M+-status (R+/M0 n = 24, R+/M + n = 3, R0/M + n = 5). The median age at diagnosis was 1.9 years (range: 0.1–17.6 years). Histologically, aCPP was diagnosed in 38 patients (40.9%) and CPP in 55 patients (59.1%). Molecular subgroup assessed by DNA methylation profiling classified three CPP patients as ”adult”, all located infratentorially, while 21 samples were assigned to the molecular subgroup “pediatric A” and 12 tumors were classified as “pediatric B” (supplemental Table 1). One patient with CPP (case no. 25, Supplemental Table 1) was diagnosed with *Li-Fraumeni* syndrome (LFS). Twelve (13%) patients experienced progressive disease (PD) of residual tumor or relapse (progression: five patients; relapse: seven patients) during follow-up. Of those, one patient with aCPP died (patient 13). Four patients (4.3%) with initial R0/M0 staging experienced relapse. Additional details on relapse and subsequent treatment are displayed by supplemental Fig. 1. Median follow-up for surviving patients was 5.5 (± 1.0) years. Upon reviewing the last follow-up of the whole cohort, the outcomes were as follows (Supplemental Table 1 “clinical courses”): CR for 82 patients (88.1%), SD for seven (7.5%), PR for two patients (2.2%), PD for one patient (1.1%) and died of disease (DOD) for one patient (1.1%).

### First-line treatment of the German CPT-SIOP registry study cohort

The clinical courses of all German CPT-SIOP Registry patients including treatment and outcomes are displayed in detail in Supplemental Table 1. During the recruitment period of the registry there were no official guidelines or recommendations. Treatment decision was discussed individually on a case-to-case basis.

### Initial surgery

Initially, all patients were treated with surgery. For three patients, the primary purpose was to only obtain a biopsy. Eighteen patients (19.4%) underwent more than one tumor surgery. Nine patients received re-surgery directly following initial incomplete resection. Of those, eight were re-operated because of residual tumours and one due to intraventricular haemorrhage.

### Postoperative adjuvant treatment

Most patients did not receive postoperative treatment (“watchful waiting”, *n* = 88, 94.6%). Five patients (5.4%) underwent postoperative adjuvant treatment with chemotherapy (R0/M + *n* = 2; R+/M + *n* = 1; R0/M0 *n* = 2). Two patients with “R0/M0” staging received chemotherapy due to the following reasons: (1) initial diagnosis of CPC (revised to aCPP by neuropathological central review) and very young age at diagnosis (case no. 2); (2) initial characterization as M+ (myelon, conus, cauda medullaris), which was subsequently rated as blood vessels by central review after start of chemotherapy (case no. 10). Retrospectively, those two patients may not have started with chemotherapy, if staging would have been correct initially. All five patients (CPP *n* = 1; aCPP *n* = 4) were in continuous CR at last follow-up evaluated by central review (median follow-up: 8.3 ± 4.6 years). No patient was irradiated during first-line treatment.

### Pattern of relapse/progression

Seven patients (7.5%) experienced relapse during the follow-up period (R0/M0 = 4, R+/M0 = 3). The median time to first relapse was 1.4 ± 3.6 (0.4–5.9) years. Of those, five patients (5.4%) were initially diagnosed with aCPP and two with CPP. Five patients (5.4%) suffered progressive disease of the residual tumor after initial surgery (R+/M0 = 4, R+/M + = 1). Those later achieved CR in two, SD in one and PR in one patient. One patient of those finally died of disease (case no. 13).

Two patients had a disseminated first relapse after complete remission (case no. 9: local relapse combined with intracranial metastasis, case no. 11: with intracranial and spinal metastases) and five patients presented local first relapse. Another patient (case no. 23) with the initial staging “R0/M0” and local first relapse had a second event later with local PD and new metastasis in the 4th ventricle.

Finally, five patients were affected by disseminated disease during their clinical course (case no. 9,11,13,14 and 23, Supplemental Tables 1 and Supplemental Fig. 1).

### Surgery at relapse/progression

Of seven patients with relapse after initial CR, six patients were re-operated once, with four achieving CR, one achieving PR and two other without relevant change in tumor volume (SD). Another two patients (case no.: 1 and 24) were re-operated once following progression of residual tumor. One patient (case no. 5) had re-surgery twice at initial staging and (third overall) re-surgery at progression. Another patient (case no. 14) received two re-surgeries after subsequent progression. This patient was also initially re-operated because of incomplete resection.

### Adjuvant treatment at relapse/progression

One patient (case no. 13) received chemotherapy and radiotherapy without re-surgery (outcome: SD), while two patients underwent re-surgery following chemotherapy and radiotherapy: one (case no. 3) after third relapse (outcome: PR) and one (case no. 15) after first relapse (outcome: CR). One patient died. This patient received six cycles of chemotherapy with alternating courses of cyclophosphamide, etoposide and vincristine (CycEV) and carboplatin, etoposide, vincristine (CarbEV) according to *CPT-SIOP-2009*,* Arm A* and local radiotherapy after progression and eventually achieved SD. He subsequently suffered from a second local relapse which was progressive over time and was finally resected (outcome: R0, but new M2b). After further dissemination was diagnosed in the spine and CSF, he received craniospinal irradiation (CSI) and achieved partial remission. He then received Sirolimus and Thalidomid (outcome: PD) and treatment was then switched to Bevacizumab. Because of vomiting, further progression of dissemination and deterioration of the facial nerve-function, the patients` family requested for interruption of therapy. The patient died 83 months after initial diagnosis.

Regarding the outcome of all study patients with relapsed/progressive disease, five subsequently achieved second CR (41.7%), three SD (25.0%), one PD (8.3%) and another two (16.7%) PR after first relapse.

### Evaluation of characteristics impacting on risk for relapse in the enlarged CPP/aCPP-cohort

**Fig. 2:**
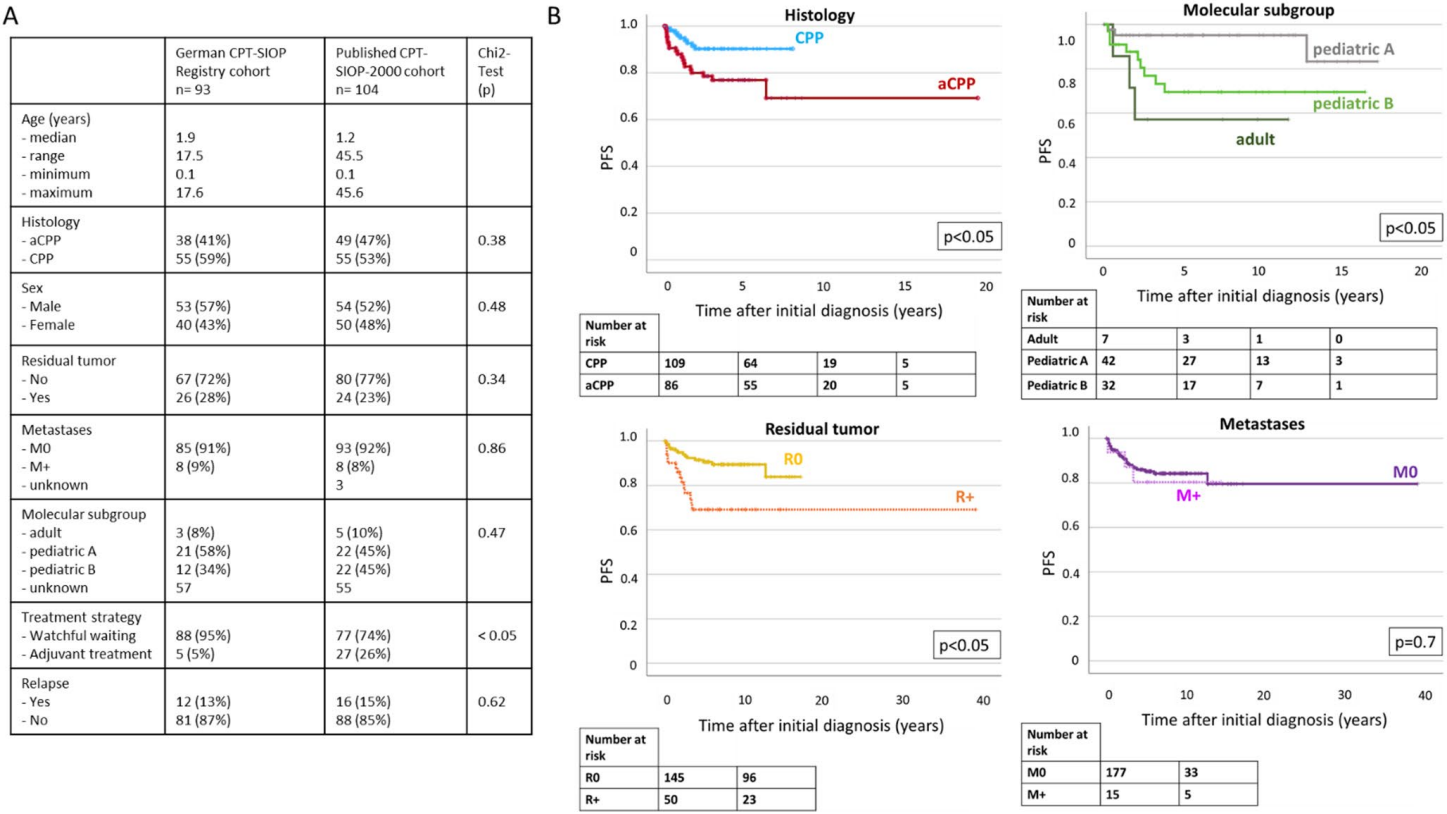
Characteristics and outcomes of the combined study cohort. **A**: Overview of demographic details of the German CPT-SIOP Registry cohort and the published CPT-SIOP-2000 cohort. **B**: Kaplan-Meier Plots: PFS according to histological papilloma type, molecular subgroup, residual tumor status and presence of metastases in the combined enlarged cohort

The enlarged cohort comprised 197 patients: *n* = 110 were diagnosed with CPP and *n* = 87 with aCPP. Molecular subgroup status was available for *n* = 85 (43.2%) patients, while information on staging and treatment was available for all cases. A difference in progression-free survival depending on histology was observed, showing an inferior PFS for patients with aCPP (Figs. 2B and 5-year PFS CPP: 90 ± 3.1, vs. aCPP: 78.6 ± 4.6, *p* < 0.05). Additionally, the residual tumor status impacted on the risk for relapse in the whole cohort (Figs. 2B and 5-year PFS R0: 90.6 ± 2.6, vs. R+: 69.11 ± 7.0, *p* < 0.05). Multivariable Cox regression for PFS including histology, molecular subgroup, residual tumor status, metastases and treatment strategy (watchful waiting vs. adjuvant chemotherapy) confirmed the impact of molecular subgroup (*p* = 0.05) and residual tumor status (*p* < 0.05, *hazard ratio* [HR] 3.6 for R + compared to R0). Notably, more patients from the CPT-SIOP-2000 cohort received adjuvant treatment compared to the registry cohort (5% vs. 26%, *p* < 0.05). This is explained by different recommendations within and after the trial (Fig. 2A). The subsequent analyses were performed separately for CPP and aCPP, to exclude bias in the results due to the histology.

### CPP: no unequivocal risk factors identified

**Fig. 3:**
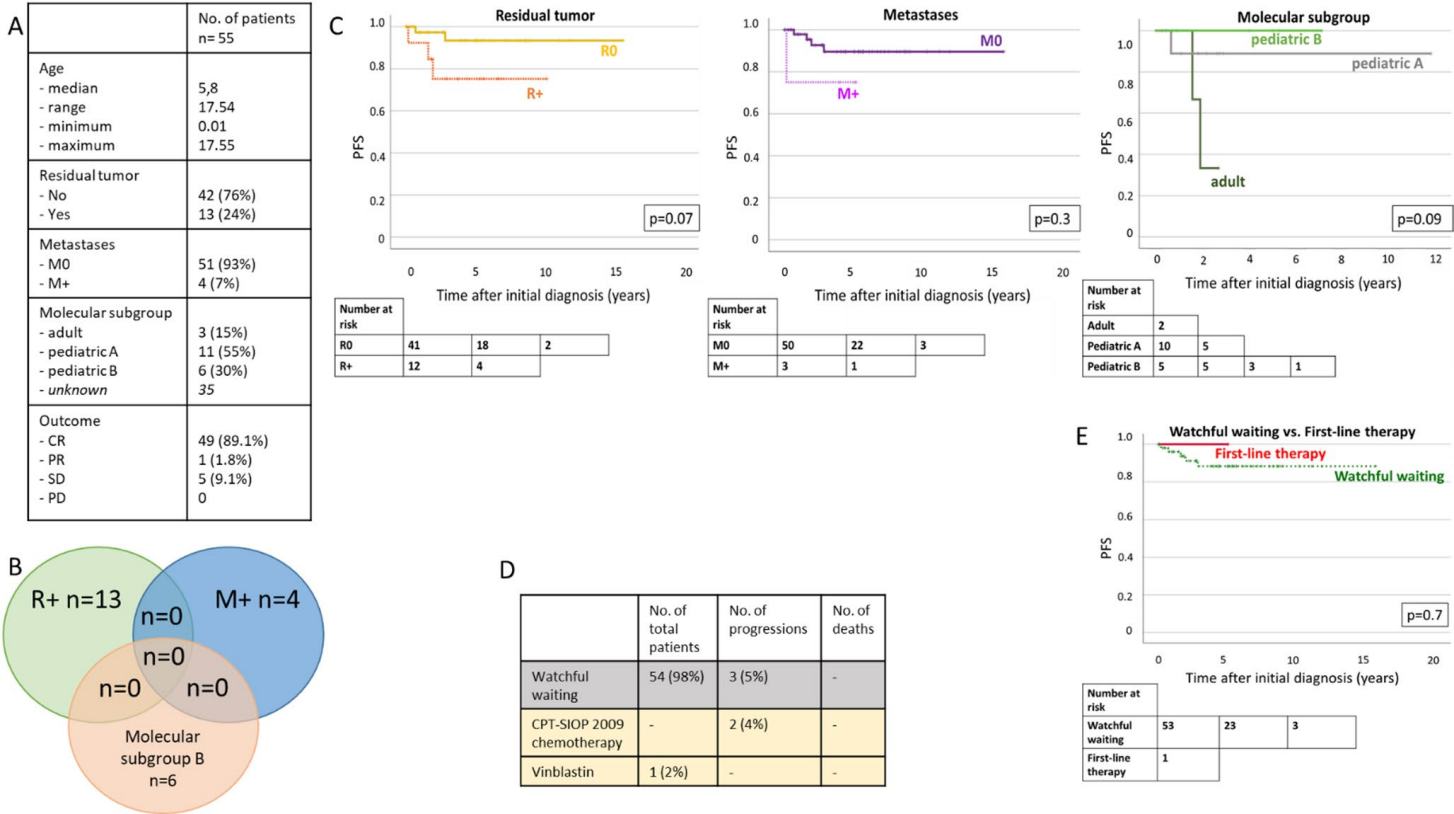
CPP– potential risk factors and outcome (German CPT-SIOP Registry cohort). **A**: Overview of characteristics. **B**: Venn-Diagram displaying overlap of potential risk factors. **C**: Kaplan-Meier Plots: PFS according to biology, residual tumor status and presence of metastases. **D**: Details of treatment strategy decisions and outcomes. **E**: Kaplan-Meier Plots: PFS according to first-line treatment decision

**Fig. 4:**
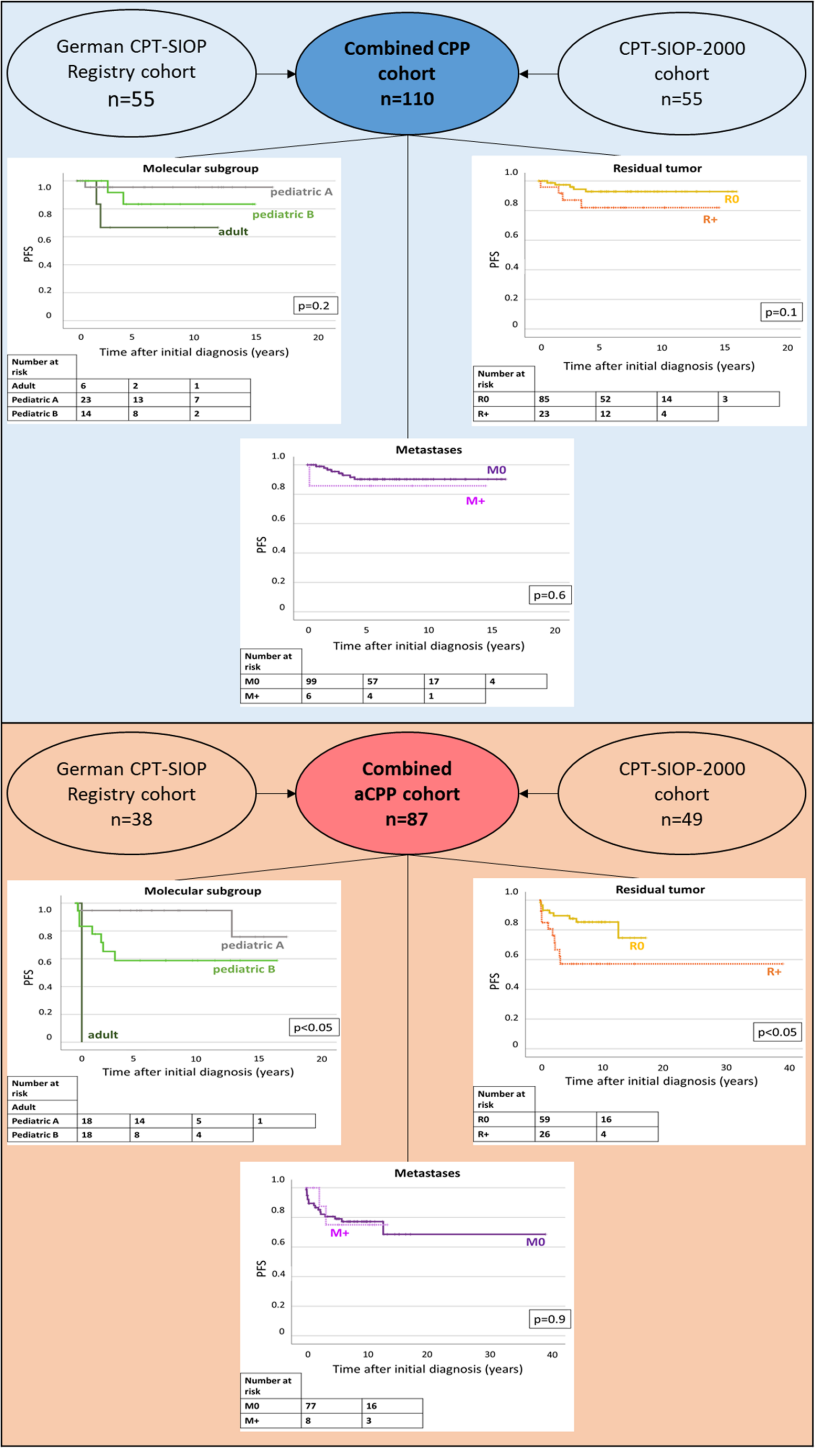
Validation of potential risk factors in combined cohort. **A**: CPP. **B**: aCPP

The details of the German CPT-SIOP Registry patients with CPP are displayed by Fig. 3A. For most patients, complete resection was achieved (76%). Metastases were rarely found (*n* = 4, 7%). Most tumors clustered with the methylation subgroup “*pediatric A*” (55%), while only one third of tumors with available subgroup clustered with “*pediatric B*” (*n* = 6, 30%). No patient died during the follow-up period of this study. Looking at potential high-risk factors (R+, M+, subgroup “*pediatric B*”) there was no overlap (Fig. 3B). Further, in the German CPT-SIOP Registry study cohort as well as in the enlarged cohort, we were unable to identify any unequivocal risk factors for relapse. Univariable analyses of residual tumor status, metastases and molecular subgroup did not show significant impact on the progression-free and overall survival (Figs. 3 and 4). Notably, a tendency towards a higher risk for relapse with residual tumor in the registry cohort (*p* = 0.07, Fig. 3C) was not confirmed in the enlarged cohort (*p* = 0.1, Fig. 4A). Furthermore, progression-free was excellent regardless of the treatment strategy (*p* = 0.7, Fig. 3E). This was also confirmed in the enlarged cohort (5-year PFS/OS watchful waiting: 90.7 ± 3.2%/100.0% vs. therapy: 87.5 ± 11.7/100.0%, p_PFS_=0.8/p_OS_=0.2). Multivariable Cox regression including molecular subgroup, residual tumor status, metastases and treatment strategy confirmed the univariable observations and did not identify any significant impact factor on neither PFS nor OS in the enlarged CPP cohort.

### aCPP: residual tumor status and molecular subgroup matter

**Fig. 5:**
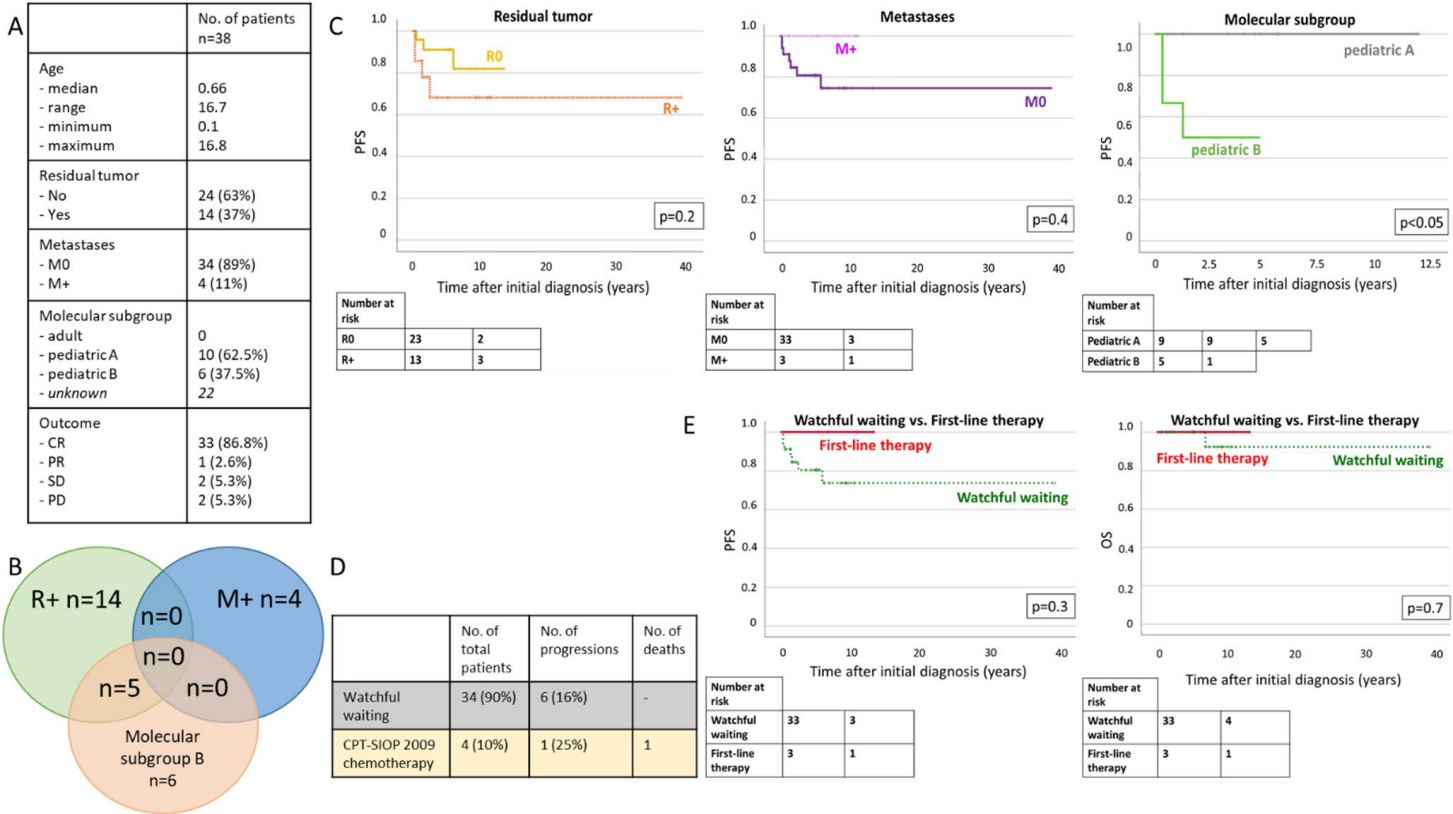
aCPP– potential risk factors and outcome (German CPT-SIOP Registry cohort). **A**: Overview of characteristics. **B**: Venn-Diagram displaying overlap of potential risk factors. **C**: Kaplan-Meier Plots: PFS according to biology, residual tumor status and presence of metastases. **D**: Details of treatment strategy decisions and outcomes. **E**: Kaplan-Meier Plots: PFS and OS according to first-line treatment decision

The details of the German CPT-SIOP Registry patients with aCPP are shown in Fig. 5A. In this cohort, 63% achieved complete resection at initial diagnosis. Dissemination was rare and found in four patients (11%). Compared to patients with CPP, aCPP clustered more often with the molecular subgroup “*pediatric B*” (37.5%). Interestingly, there was an overlap between the molecular subgroup “*pediatric B*” and the presence of residual tumor, but not metastases (Fig. 3B). In the registry cohort, univariable analyses revealed a significant inferior PFS for patients with molecular subgroup “*pediatric B*” (5-year PFS “pediatric B”: 50.0% vs. “pediatric A”: 100.0%, *p* < 0.05), while residual tumor status, presence of metastases and treatment strategy did not impact on PFS/OS (Fig. 4C + E). In the enlarged aCPP cohort, the impact of the molecular subgroup was also significant showing a lower PFS for pediatric B (5-year PFS pediatric A: 94.7 ± 5.1 vs. pediatric B: 58.7 ± 12.15%, *p* < 0.05, Fig. 4). Further, an inferior PFS was observed for patients with residual tumor (5-year PFS R+: 57.1 ± 10.5% vs. R0: 87.5 ± 4.4%, *p* < 0.05), but not for the presence of metastases. Multivariable Cox regression confirmed this observation (HR R + compared to R0: 3.4, *p* < 0.05). Still, this impact was not seen in the OS analysis (HR R + compared to R0: 1.1, *p* = 1.0). In the enlarged cohort, patients receiving postoperative chemotherapy (*n* = 23) did not benefit compared to patients with watchful waiting strategy (*n* = 64; 5-year PFS/OS watchful waiting: 79.6 ± 5.3%/100.0% vs. therapy: 78.3 ± 8.6/91.3 ± 5.9%, p_PFS_=0.5/p_OS_=0.2). In the enlarged cohort, multivariable Cox regression for PFS including, molecular subgroup (pediatric A and pediatric B), residual tumor status and treatment strategy (watchful waiting vs. adjuvant chemotherapy) confirmed the significant impact of residual tumor status (*p* < 0.05, *hazard ratio* [HR] 7.9 for R + compared to R0). Multivariate cox regression showed no impact of molecular subgroup. The presence of metastases was not included into the model due to the low number of metastatic aCPP and missing impact in the univariate analysis.

### Impact of chemotherapy in patients with high-risk aCPP

Patients with aCPP and residual tumor and / or molecular subgroup pediatric B were analysed separately to evaluate the impact for adjuvant chemotherapy. Of those patients seven received adjuvant chemotherapy, while eight were subjects to watchful waiting. Univariate Kaplan-Meier analyses showed no difference of PFS for patients with aCPP and both R + and pediatric B.

## Discussion

In this study, we present a large cohort of previously unpublished clinical courses of pediatric patients with CPP and aCPP, drawn from the German CPT-SIOP Registry and supplemented by cases from the previously published CPT-SIOP-2000 trial reported by Wolff et al. [1] The patients` characteristics, including median age at diagnosis and sex distribution are consistent with findings from previous studies [15, 16]. Unlike earlier analyses that required the exclusion of cases due to initial misdiagnoses, our dataset included only a single case where diagnosis was revised to a lower WHO grade following central pathology review [16, 17]. Additionally, the extended median observation time of 5.5 years and the availability of detailed treatment data underscore the robustness and clinical relevance of our findings.

Our study confirms and underlines the presumption that the presence of residual tumor impacts on the risk of relapse in aCPP, but not in CPP. This is in line with previous reports [7, 18–20]. Further, Chen described in a serial case report of nine aCPP, that the initial tumor volume measured by MRI was significantly higher and larger in residual tumors as well as intraoperative bleeding was more likely compared to CPP [21]. Still, there are also reports suggesting that long-term follow up without progression of a residual tumor may eventually be observed [18, 22]. Apart from the impact of residual tumor on PFS, an impact on OS was not observed in our cohort. This observation may be explained by successful use of combinations of re-surgery, chemotherapy and irradiation at relapse. Other characteristics associated with inferior PFS in aCPP patients reported are an age > 2 years at initial diagnosis and clustering with the molecular subgroup “*pediatric B.* [1]

Thomas et al. reported that mitotic count was not associated with prognosis in children under 3 years old, which explains the favourable outcomes of aCPP in this age group [13]. aCPPs within methylation cluster 3 (pediatric CPP, aCPP, and CPC of supratentorial location) showed frequent progression, while no tumor progression was observed in aCPPs from methylation cluster 1 (pediatric CPP and aCPP predominantly of supratentorial location) [7]. In line with this, we observed an inferior PFS for patients with aCPP subgroup “*pediatric B*” in the registry cohort, with statistically significance in the enlarged aCPP cohort. Interestingly, there was an overlap between the molecular subgroup “*pediatric B*” and the presence of residual tumor. This may suggest more difficult surgical condition for these tumors, but may also point to potentially confounding effects.

Apart from the impact of residual tumor and molecular subgroup, the presence of dissemination has been used as a high-risk characteristic in aCPP indicating adjuvant treatment [1, 11]. Nevertheless, we were not able to confirm that metastases at initial diagnosis is associated with inferior PFS and OS. While some authors recommended resecting metastases or have reported successful outcomes from metastatic resection, the adequacy of surgical treatment without adjuvant therapy for effective tumor control remains uncertain [21, 22]. Further, Passariello described two patients, who were successfully cured with surgery alone, while the others two experienced recurrent disease [23]. 

Most reports do not include biological information. Therefore the management of subtotal resected aCPPs continues to be debated. Chemotherapy demonstrated efficacy in enhancing survival rates in cases of incompletely resected, metastatic, and recurrent aCPP [11, 24–26]. Tavallaii et al. observed significant reduction in radiologic relapse and postoperative tumor dissemination/metastasis rates in patient with aCPP and residual tumor following adjuvant therapies [27]. Siegfried et al. also observed favourable outcomes for patients with incomplete resected aCPP following adjuvant chemotherapy [17]. Consistently, we did not observe any disease progressions following adjuvant treatment. Still, pursuing watchful waiting was not associated with an inferior PFS and OS. Taking these findings into account, it has to be carefully scrutinise, if the indication of adjuvant treatment can be derived from the isolated presence of residual tumor in aCPP patients, but rather by including information on age and molecular subgroup as well.

Secondly, we demonstrated a high rate of complete resection achieved in most patients with CPP and subsequently excellent outcomes. In line with various previously published studies, completely resected CPP are typically associated with a favourable outcome without the need for adjuvant therapy [1, 16, 17, 19, 20, 26–28]. Univariable analyses of potential risk factors remained unremarkable in our study. Notably, a trend toward a higher risk of relapse associated with residual tumor in the registry cohort was not corroborated by findings from the expanded cohort.

Also previous studies suggest, that tumor size is not a significant prognostic factor [26]. Still, for tumors located in the fourth ventricle, where complete resection is challenging, adjuvant therapy following surgery can be employed as a second-line treatment to reduce the risk of relapse [15]. While metastases being rare, consistent with other studies, minority of tumors with available subgroup data cluster with “*pediatric B*”. Notably, we observed no overlap among potential high-risk characteristics. In this study, PFS and OS of patients with CPP remained favourable irrespective of treatment strategy (watchful waiting vs. chemotherapy) underlining that patients with CPP can be fully cured through surgical resection, assuming complete resection, especially considering the prevention of side effects of radiochemotherapy.

Overall, the management of CPP/aCPP recurrence and the role of irradiation and its timing remains unclear.

In the German Registry cohort, four patients were irradiated after relapse/progressive disease, all of them with aCPP, while receiving differing outcome. Other retrospective studies proved efficacy of irradiation [11, 29]. Nevertheless, in consideration to the risk of late neurological sequelae, radiotherapy should be administered to only a limited number of patients.

Apart from these observations, this retrospective study has limitations, especially regarding the overall limited number of cases explained by the rarity of the disease itself. Especially the limited number of patients receiving chemotherapy must lead to a restrictive interpretation of the findings. Additionally, the limited availability of molecular data hampers the interpretation of the results. Especially, observations in a multivariate context must be interpreted with caution. Moreover, exploratory evaluation of additional biological characteristics as *TP53* mutations was not possible. Future studies are necessary to comprehensively assess the impact of the molecular characteristics.

Finally, the present study reports on a large, unpublished cohort of German patients diagnosed with CPP and aCPP combined with previously published cases. Notably, histological status emerged as a strong predictor of PFS. Despite the rarity of postoperative adjuvant treatment, chemotherapy demonstrated efficacy on aCPP, particularly in cases of incomplete resection. Comprehensive information on all three, molecular subgroup, clinical and histopathological features are required to identify objective markers to enable robust determination of risk for disease progression or relapse. Larger series of patients with choroid plexus tumors across all age with comprehensive biological data in addition to histopathology and clinical information need to be analysed and may provide further useful prognostic information. aCPP patients with high risk constellation showed no tendency towards lower PFS. Based on this, it appears reasonable to use postoperative chemotherapy in selected cases of aCPP with molecular subgroup “*pediatric B”* and residual tumor, given the good outcome after treatment, as well as at relapse.

## Electronic supplementary material

Below is the link to the electronic supplementary material.

Supplementary Material 1: **Table 1** “clinical courses”

Supplementary Material 2: **Fig. 1** “patients with relapse or progression - Treatment and outcome”

## Data Availability

The datasets generated during and/or analysed during the current study are available from the corresponding author on reasonable request.
