# Supplementary figures and images for: CPT-SIOP registry: evaluation of risk factors for disease progression in pediatric choroid plexus papilloma
Source: J Neurooncol. 2025 Jun 26;175(1):333–43. doi: 10.1007/s11060-025-05136-4 (PMC12367813; doi:10.1007/s11060-025-05136-4)

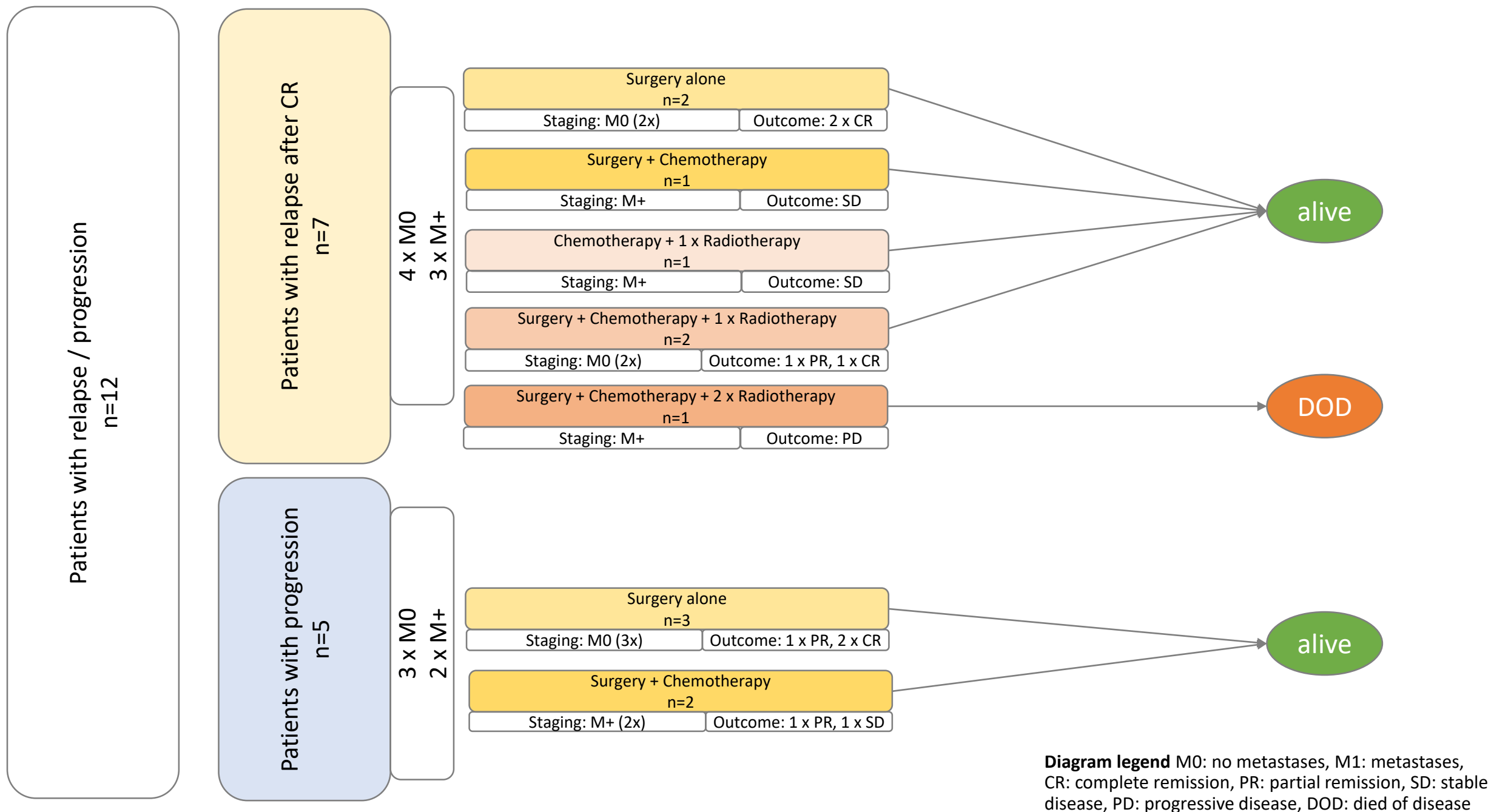

Supplement: Supplementary file 2 — Supplementary Material 2: Fig. 1 “patients with relapse or progression - Treatment and outcome” [file 11060_2025_5136_MOESM2_ESM.pdf]
